# Incidence and risk factors for postpartum hemorrhage among transvaginal deliveries at a tertiary perinatal medical facility in Japan

**DOI:** 10.1371/journal.pone.0208873

**Published:** 2019-01-09

**Authors:** Tatsuya Fukami, Hidenobu Koga, Maki Goto, Miho Ando, Sakiko Matsuoka, Atsushi Tohyama, Hiroko Yamamoto, Sumie Nakamura, Takahiro Koyanagi, Yoko To, Haruhiko Kondo, Fuyuki Eguchi, Hiroshi Tsujioka

**Affiliations:** 1 Department of Obstetrics and Gynecology, ASO Iizuka Hospital, Iizuka, Fukuoka, Japan; 2 Department of Health Information Management, ASO Iizuka Hospital, Iizuka, Fukuoka, Japan; 3 Supporting Unit of Medical Research, ASO Iizuka Hospital, Iizuka, Fukuoka, Japan; University of North Carolina at Chapel Hill, UNITED STATES

## Abstract

Postpartum hemorrhage (PPH) remains a leading cause of maternal death worldwide, and it is important to understand the relative contributions of different risk factors. We assessed the incidence of these among cases of transvaginal delivery. Between June 2013 and July 2016, a prospective cohort study was conducted at a tertiary perinatal medical facility in Japan. Women were administered a questionnaire to ascertain risk factors for PPH, defined as a blood loss of 1,000 ml or more assessed using a calibrated under-buttocks drape and collection vessel at childbirth. We analyzed 1,068 transvaginal deliveries of singleton pregnancies. The incidence of PPH was 8.7%, and of severe PPH (1,500 ml blood loss or more) was 2.1%. Risk factors for postpartum hemorrhage among the deliveries were: fetal macrosomia (over 4000 g); pregnancy-induced hypertension; pregnancy generated by assisted reproductive technology; severe vaginal or perineal lacerations; and weight gain over 15 kg during pregnancy. Such high weight gain significantly increased the incidence of PPH compared with women showing less than 10 kg weight gain during pregnancy. Monitoring these identified risk factors could enable extra vigilance during labor, and preparedness for managing PPH in all women giving birth.

## Background

Postpartum hemorrhage (PPH) is defined more than 500 ml of blood bleeding following vaginal delivery [1]. PPH is considered severe when blood loss exceeds 1,000 ml after a vaginal delivery, or results in signs or symptoms of circulating blood volume instability [1]. It is a major cause of maternal mortality especially in developing countries and is the cause of 25% of maternal deaths worldwide [2]. It is the most common maternal morbidity even in highly resourced countries and is increasing in incidence [3]. Sequelae of PPH include hypotension, anemia, and fatigue, which can make breastfeeding and maternal care of the newborn more difficult [4]. The Society of Obstetricians and Gynaecologists of Canada has published guidelines on the prevention and management of this complications [5]. They summarize the causes for PPH as related to abnormalities of one or more of four basic processes, namely the “four Ts”: tone, trauma, tissue, and thrombin. Atonic bleeding is major factor of PPH. Risk factors include antepartum and intrapartum conditions as including a history of PPH, multiple pregnancies, fetal macrosomia, primigravida, grand multiparity, older age, preterm births, genital tract injuries, non-use of oxytocin for PPH prophylaxis, labor induction, cesarean delivery and intra-uterine fetal deaths [1, 6–9]. However, 20% of patients who develop PPH have no risk factors, so providers must be prepared to treat it at every delivery [10]. There is little information on the magnitude and risk factors for PPH. Common causes include uterine atony, trauma including genital tract injuries, placental retentions and failure of the blood coagulation system. Uterine atony is responsible for the majority (75%) of cases of PPH [11]. To obstetrics providers, risk factor identification in the antenatal and intrapartum periods might enable timely interventions to prevent PPH. This study was undertaken to assess the incidence of, and risk factors for PPH among transvaginal deliveries at a tertiary perinatal medical facility in Japan.

## Methods

**Fig 1:**
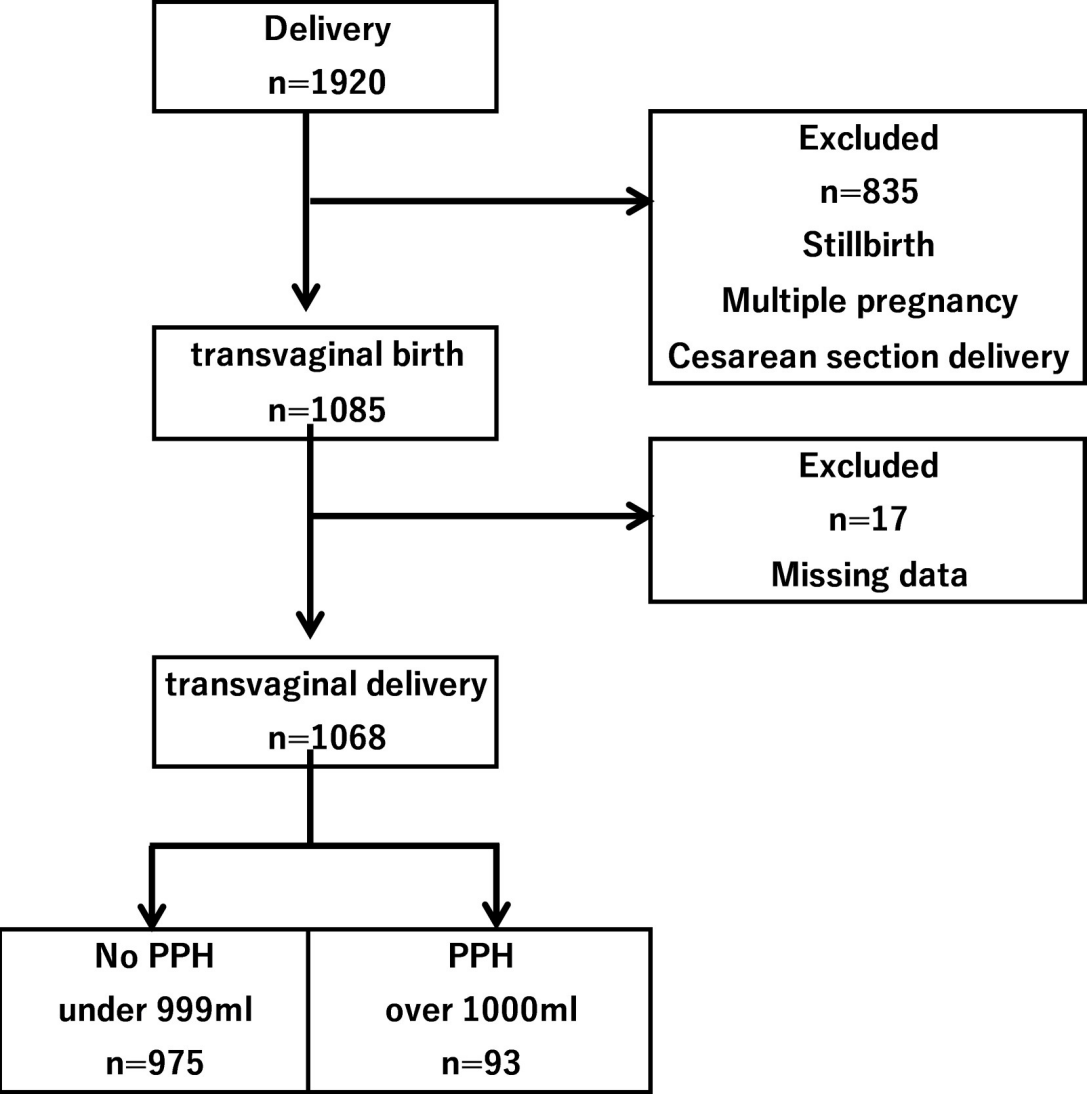
Profiles of the study participants.

Our medical center is one of the tertiary perinatal medical facilities in Japan. Ethical approval was obtained from the ASO Iizuka Hospital, Iizuka, Japan. Informed written consent was obtained from all participants at enrolment. Between June 2013 and July 2016, a prospective cohort study was conducted. Women were administered a questionnaire to ascertain risk factors for PPH, defined as a blood loss of 1,000 ml or more at childbirth. PPH is defined as the blood loss of more than 500 ml within the first 24 hours following childbirth. In this study, we defined as the blood loss of more than 1,000 ml which influences results in signs or symptoms of circulating blood volume instability. Pregnant women were recruited at 22 weeks of gestation or greater. The study excluded women who underwent a caesarean-section delivery, or who had a stillbirth or a multiple pregnancy. Cases with data missing for the primary outcome of blood loss were excluded from the analysis (Fig 1). The health providers in the delivery rooms in these health facilities were trained in the data collection procedure and on the measurement of postpartum blood loss. During enrolment, interviewer-administered questionnaires were used to collect data on risk factors including maternal age, parity, maternal body weight and body mass index (BMI) before pregnancy, and weight gain during pregnancy. Gestational age at birth was calculated based on ultrasound scan estimations or on the mother’s recollection of her last normal menstrual period. The research team noted whether labor was induced or augmented with oxytocin, the mode of delivery (normal or assisted vaginal delivery), and any severe vaginal/perineal lacerations. The primary outcome was PPH defined as a blood loss of 1,000 ml or more after childbirth. If the blood loss is over 100ml, the vital sign deteriorated easily, so we defined more than 1000ml hemorrhage as PPH. The total blood loss collected in the calibrated conical receptacle was established by the attending midwife. The following variables were collected from medical record and questionnaire: maternal age; parity; the use of assisted reproductive technology (ART); maternal smoking habit; pregnancy-induced hypertension (PIH); maternal body weight and BMI before pregnancy; labor induction/augmentation by oxytocin; assisted vaginal delivery; severe vaginal/perineal lacerations; and neonatal birthweight. Multivariate Logistic regression analysis was used to estimate odds ratios (ORs) and 95% confidence intervals (CIs) and control for the potential confounders. Explanatory variables included in this model were those that statistically significant for outcome in the univariate analysis. However, we did not include gestational week and birth weight simultaneously in this model because the correlation between them was strong (Pearson correlation coefficient, r = 0.672, p<0.001). We did not include maternal age, maternal height, gestational age at delivery, weight gain during pregnancy, use of ART, PIH and severe vaginal/perineal lacerations were used for explanatory variables. Two-sided P values of < 0.05 were regarded as statistically significant. All analyses were performed using SAS software version 9.4 (SAS Institute, Inc., Cary, NC, USA). This study was approved by the institutional review board (IRB) of ASO Iizuka Hospital, Fukuoka, Japan.

## Results

**Fig 2:**
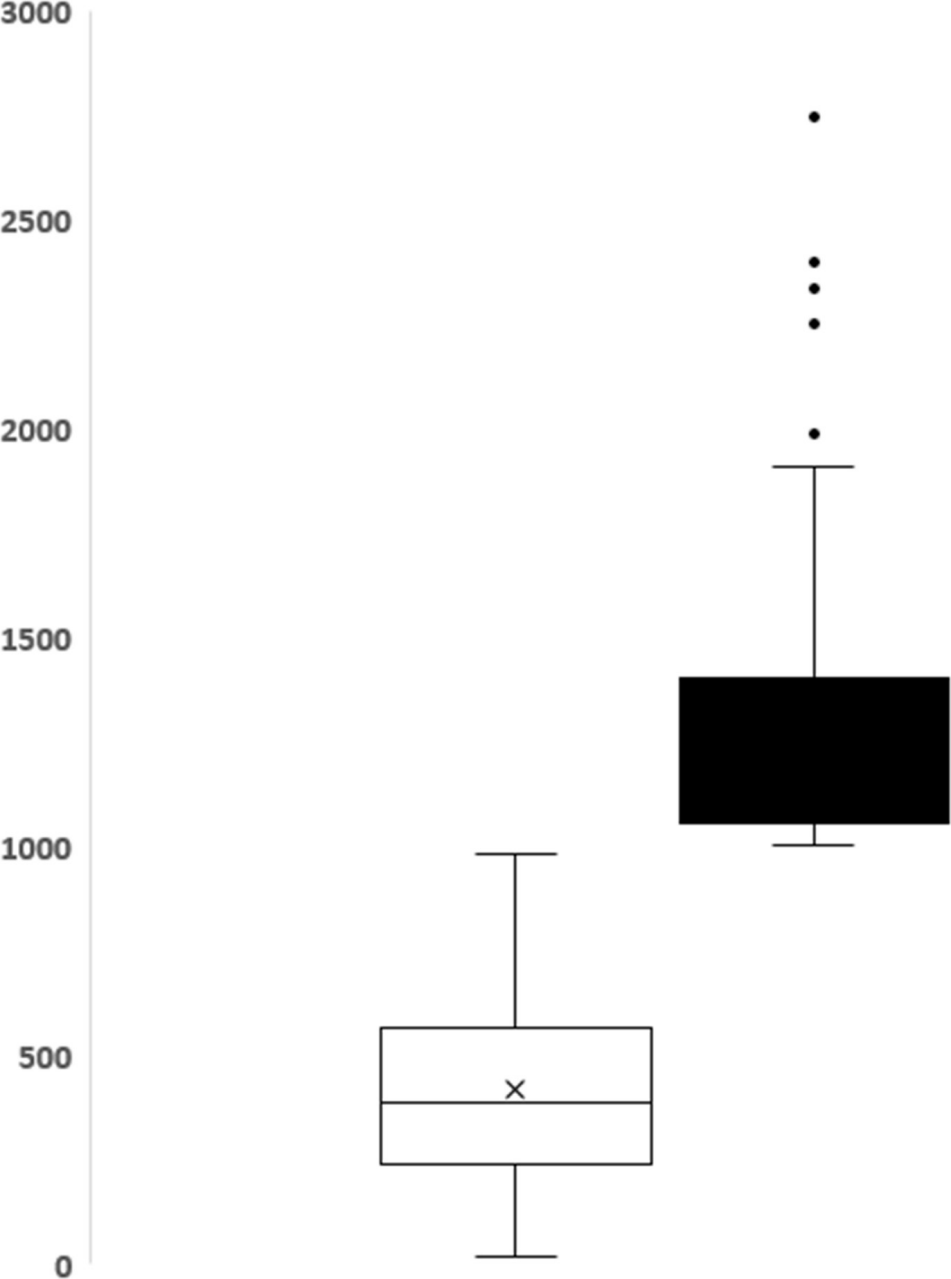
The median and interquartile range of blood loss.

Fig 2 shows the median and interquartile range of blood loss. Table 1 shows the distribution of sociodemographic, antepartum and intrapartum factors of the study participants, and their association with PPH.

**Table 1:** Characteristics of the study population (N = 1068).

	No PPH (n = 975)	PPH (n = 93)	Total (N = 1068)	minimum-maximum
Maternal age at delivery	30.4 ± 6.0	30.4 ± 6.0	30.4 ± 6.0	(15–44)
Parity	0.8 ± 1.1	0.9 ± 1.1	0.8 ± 1.1	(0–6)
Pre-pregnant body weight (Kg)	53.5 ± 10.9	54.1 ± 8.3	53.5 ± 10.7	(32–105)
Pre-pregnant body mass index	21.6 ± 4.1	21.7 ± 3.3	21.6 ± 4.1	(14.6–41.2)
Weight gain during pregnancy (Kg)	9.0 ± 5.4	10.8 ± 4.3	9.2 ± 5.3	(-10.3–45.5)
Gestational week at delivery	38.7 ± 2.2	39.3 ± 1.3	38.8 ± 2.1	(23.1–42.3)
Blood loss (ml)	425 ± 226	1,344 ± 386^a^	505 ± 356	(40–2745)
Neonatal birth weight (g)	2,855 ± 511	3,237 ± 421^a^	2,884 ± 515	(580–4596)

^a^Significantly different from No PPH group.

The results are described by medians and interquartile ranges. Data collected among 1,068 women showed a mean blood loss of 505 ± 356 ml and ranged from 40 to 2,745 ml. Overall, 93 (8.7%) women had PPH (1,000 ml or more) and 22 (2.1%) had severe PPH (1,500 ml or more; Table 2).

**Table 2:** Prevalence of PPH.

Blood loss (ml)	N = 1068	%
less 499	648	60.7
500–1000	327	30.6
1000–1500	71	6.6
over 1500	22	2.1

The use of ART, excessive weight gain (over 15 kg) during pregnancy, complicated PIH, severe vaginal/perineal lacerations and having a macrosomic baby were contributing factors for PPH (Table 3).

**Table 3:** Association between the risk factors and postpartum hemorrhage.

Variable		No PPH n = 975 (%)	PPH n = 93 (%)	*P* value
Age at birth				
	under 19	51 (5.2)	3 (3.2)	<0.05
	20–35	652 (66.9)	62 (66.7)	Reference
	35–40	228 (23.4)	21 (22.6)	*NS*
	over 40	44 (4.5)	7 (7.5)	<0.05
Pre-pregnant body mass index				
	under 18.4	173 (17.7)	18 (19.4)	*NS*
	18.5–24.9	661 (67.8)	59 (63.4)	Reference
	over 25.0	141 (14.5)	16 (17.2)	*NS*
Parity				
	Primipara	485 (49.7)	44 (47.3)	Reference
	Multipara	490 (50.3)	49 (52.7)	*NS*
Artificial reproductive technique				
	No	945 (96.9)	84 (90.3)	Reference
	Yes	30 (3.1)	9 (9.7)	<0.01
Smoking habit				
	No	911 (93.4)	86 (92.5)	Reference
	Yes	64 (6.6)	7 (7.5)	*NS*
Weight gain during pregnancy (Kg)				
	less 9.9	552 (56.6)	36 (38.7)	Reference
	10.0–14.9	337 (34.6)	40 (43.0)	*NS*
	over 15.0	86 (8.8)	17 (18.3)	<0.01
Gestational week at delivery (weeks)				
	after 40/0	283 (29.0)	31 (33.3)	*NS*
	37/0-39/6	549 (56.3)	56 (60.2)	Reference
	before 36/6	143 (14.7)	6 (6.5)	<0.01
Pregnancy induced hypertension				
	No	913 (93.6)	78 (83.9)	Reference
	Yes	62 (6.4)	15 (16.1)	<0.01
Labor induction/ augmentation by oxytocin				
	No	657 (67.4)	60 (64.5)	Reference
	Yes	318 (32.6)	33 (35.5)	*NS*
Assisted vaginal delivery				
	No	897 (92.0)	84 (90.3)	Reference
	Yes	78 (8.0)	9 (9.7)	*NS*
Severe vaginal/perineal laceration				
	No	834 (85.5)	68 (73.1)	Reference
	Yes	141 (14.5)	25 (26.9)	<0.01
Neonatal birth weight (g)				
	less 2499	209 (21.4)	2 (2.2)	<0.01
	2500–3499	679 (69.6)	67 (72.0)	Reference
	3500–3999	77 (7.9)	20 (21.5)	<0.05
	over 4000	10 (1.0)	4 (4.3)	<0.01

Table 4, lists the factors associated with increased risk for PPH from the logistic regression analysis. These included the use of ART (OR 3.479; 95% CI 1.47–8.24); PIH (OR 3.159; 95% CI 1.65–6.06) and severe vaginal/perineal lacerations (OR 1.978; 95% CI 1.19–3.31).

**Table 4:** Adjusted odds ratio and corresponding 95% confidence intervals for delivery related outcomes.

	Odds ratio	95% Confidence interval
Artificial reproductive technique pregnancy	3.48	1.47–8.24
Pregnancy induced hypertension	3.16	1.65–6.06
Severe vaginal/perineal laceration	1.98	1.12–3.31

All outcomes are adjusted for maternal age, maternal height, gestational age at delivery, weight gain during pregnancy, artificial reproductive technique pregnancy, pregnancy induced hypertension, severe vaginal/perineal laceration.

## Discussion

The overall incidence of PPH was 8.7% and that of severe PPH was 2.1%. The risk factors for PPH were the use of ART, PIH, severe vaginal/perineal lacerations and having a macrosomic baby. The incidence of PPH in this study was higher than that reported previously. Sosa et al. reported that 10.8% of woman lost more than 500 ml and 1.9% lost greater than 1,000 ml [12]. Calvert et al. reported that the prevalence of PPH (blood loss >500 ml) ranged from 7.2% in Oceania to 25.7% in Africa [13]. The prevalence of severe PPH (blood loss >1,000 ml) was highest in Africa at 5.1% and lowest in Asia at 1.9%. This high incidence of PPH in our study may have been influenced by the characteristics of the study population. Our hospital is a single tertiary perinatal medical facility in Japan that is reported to have higher rates of PPH. We excluded cesarean deliveries and multiple pregnancies in this study as the former associated with an increased risk of PPH [14,15]. Multiple pregnancies are also associated with an increased risk of PPH [15,16]. Singleton transvaginal deliveries were analyzed in this study to identify the risk factor clearly. The risk for PPH was highest for women using ART. This is consistent with previous studies [17,18]. Thus, Zhu et al. reported that placental adherence occurred more frequently in a group of women after using ART.^17^ Placental adherence reflects abnormal development, and is an independent risk factor for PPH. However, there was no placental adhesion or uterine inversion in our study. Another possibility is the presence of uterine myomas or uterine anomalies, but we did not investigate maternal uterine factors. Complicated PIH was the second major risk factor in our study. Hematological abnormalities can develop in some women with PIH and the levels of plasma clotting factors may decrease. Other possible and not registered confounders were the use of magnesium sulfate, increased blood loss via vasodilatation, a tocolytic effect predisposing to uterine atony, prolonged bleeding time through inhibition of platelet activity and red cell deformities [19]. Lacerations and hematomas resulting from birth trauma can cause significant blood loss that can be lessened by hemostasis and timely repair. Sutures for hemostasis should be placed if direct pressure does not stop the bleeding. Episiotomy increases blood loss as well as the risk of anal sphincter tears and should be avoided unless urgent delivery is necessary and the perineum is felt to be a limiting factor in achieving delivery [20]. Additionally, it was clear from our study that excessive weight gain during pregnancy was associated with an increased risk of PPH. Excessive weight gain can lead to a macrosomic baby, which will overdistend the uterus and uterine atony. There is no data, if macrosomia inducted earlier to prevent the PPH.

This study had limitations. First, our data set included only Japanese women, and it is unclear whether the results can be extrapolated to women of other ethnic groups. Second, we are in a tertiary obstetrics center. This might have introduced selective bias in the patient background characteristics. Third, this study did not include maternal uterine factors. Fourth, we did not investigate the duration of labor and HELLP syndrome associated with PIH.

In conclusion, the risk factors for PPH in our setting were the use of ART, PIH, severe vaginal/perineal lacerations and macrosomic neonates. Extra vigilance during the antenatal and peripartum periods is needed to identify women at risk and enable early intervention to prevent PPH. It is important to remember that we need to prepare for PPH in all women giving birth, as some develop it without any known risk factors.

## Supporting information

S1 File Database of each parameter for this manuscript. (XLSX)
